# PubMed QUEST: The PubMed Query Search Tool. An informatics tool to aid cancer centers and cancer investigators in searching the PubMed databases

**Published:** 2007-02-12

**Authors:** David A. Hanauer, Arul M. Chinnaiyan

**Affiliations:** 1 Department of Pediatrics,; 2 Department of Pathology,; 3 Comprehensive Cancer Center Bioinformatics Core, University of Michigan Medical School, Ann Arbor, MI 48109, USA

**Keywords:** PubMed Databases, Bibliographic Medical Informatics Applications, Information Storage and Retrieval

## Abstract

Searching PubMed for citations related to a specific cancer center or group of authors can be labor-intensive. We have created a tool, PubMed QUEST, to aid in the rapid searching of PubMed for publications of interest. It was designed by taking into account the needs of entire cancer centers as well as individual investigators. The experience of using the tool by our institution’s cancer center administration and investigators has been favorable and we believe it could easily be adapted to other institutions. Use of the tool has identified limitations of automated searches for publications based on an author’s name, especially for common names. These limitations could likely be solved if the PubMed database assigned a unique identifier to each author.

## Introduction

Cancer centers around the nation must constantly assess the progress made in their research endeavors. A very important component of both the core grant renewal process, and the ability to measure the interim progress of research in each program, is the determination of not only the total number of publications produced but the collaborations that occurred in the process of creating each publication. As cancer research becomes ever more complex, cancer centers that foster a variety of collaborations among disparate groups will likely rise to the forefront as leaders in translational research.

Our institution, like many others, is divided into a subset of similar research fields referred to as core programs. Each core program receives funding from various sources including a substantial portion from the National Cancer Institute as part of a core-grant renewal process that occurs on a five-year cycle.

Cancer Center core members are assigned to core programs related to their area of research in order to enhance collaborations among investigators. Such collaborations are referred to as either intra-programmatic or inter-programmatic depending on the programs to which the authors of each publication belong. It has been our experience that the definitions used to denote intra- and inter-programmatic publications have varied among individuals and the consistency with which papers are classified are not reliable. Additionally, reporting the publications has often been the role of each program leader, who either has had to aggregate the publications that each program member reports or has to resort to the tedious process of searching PubMed manually.

PubMed is a search engine created by the National Library of Medicine to aid in the rapid searching and retrieval of articles related to medicine and the life sciences (NCBI, 2005a). The tool offers many powerful features, including Boolean operators and the ability to focus a search using “Search Field Descriptions and Tags” (NCBI, 2005b). Such tags allow searches to be filtered based on a wide variety of options including the institution of the primary author, the dates of publication, and specific medical subject heading (MeSH) topics. Unfortunately, the formation of these complex queries is beyond the reach of the typical cancer center investigator who has neither the expertise to form such queries nor the time to learn how. In fact, our institution actually offers continuing medical education credit for individuals who take classes in the use of PubMed.

Multiple applications and interfaces have been written to assist in searching PubMed, often for automated text-mining purposes, but also to simply retrieve references based on user-generated queries (Ding & Berleant, 2005; Lin et al., 2004; Gall & Brahmi, 2004). However, the process of identifying articles of interest can still be quite complex and labor intensive. For our most recent grant renewal cycle we chose to improve this process by creating an intuitive tool to aid in searching, filtering, aggregating, and marking publications of interest to the cancer center. A description of this tool, the PubMed Query Search Tool (QUEST) follows.

## Methods

We created PubMed QUEST using freely available open-source tools that will run on multiple operating systems. It was built using the hypertext markup language (HTML), JavaScript, and Java Server Pages.

**Figure 1.**
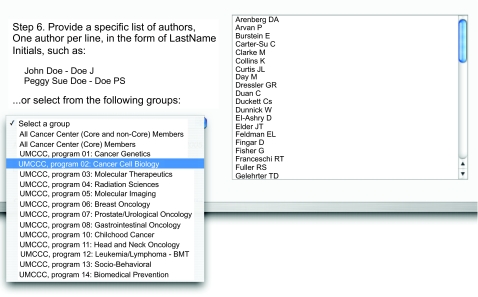
A screen shot of the section of the tool allowing users to choose from pre-made listed of authors, grouped by program.

During the testing phase, input from users was utilized to add desirable features in order keep the application simple, yet powerful. The tool consists of a web front-end with which the user interacts. The tool is divided into eight logical steps and users simply need to work through each step to obtain the final results. Each step allows the user to customize specific aspects of the desired parameters for the search query. These options include the ability to limit the search based on: (1) a date range; (2) a specific institution using suggested keywords; (3) specific topics as defined in MeSH; (4) specific journals; and (5) specific authors (Figure 1).

Formatting options are also available including the option to denote articles representing both intra-and inter-programmatic collaborations as well as formatting the names of cancer center investigators in bold. Pre-defined drop-down menus were created so that groups of journals could be rapidly selected. In addition, pre-defined menus were created so that authors could also be grouped by program without the need to enter the names manually. A help button was made available for each step in case users had difficulty.

In order to denote intra- and inter-programmatic collaborations for cancer center programs, we developed a strict definition of each that could be consistently applied across the cancer center. These definitions are:

### Intra-programmatic collaboration:

For the program of interest, an intra-programmatic collaboration is where at least two authors in the publication are part of that program.

### Inter-programmatic collaboration:

For the program of interest, an inter-programmatic collaboration is where, among the authors in the publication who are cancer center members, there exists at least one pair of authors who do not share any overlap between the programs to which they belong.

Once a user specifies the correct parameters for the search query and presses the “Submit Query” button, PubMed QUEST formulates the proper query based on the user’s input. Often, multiple queries are created based on the input. These queries are sent in succession to PubMed and the results are obtained in the extensible markup language (XML) format. The XML results are parsed using the open source Java Document Object Model (JDOM™) and further processed using Java. The results are then sent via e-mail to the user in an easy to read format that can be copied and pasted into a word processing document. Searching and the subsequent processing generally requires anywhere from a few seconds to 30 minutes depending on the complexity of the information requested.

## Discussion

Our experience with PubMed QUEST has been favorable. Our application provides for an intuitive interface to PubMed that also incorporates local data relevant to our cancer center such as the program membership lists and the lists of high-impact journals relevant to each program. Use of the tool allows us to answer many questions rapidly such as “How many high impact publications did the breast core program publish within the last 4 years?” The consistency with which publications are formatted also saves a considerable amount of time when trying to aggregate the results from the multiple programs that comprise our cancer center.

PubMed QUEST directly queries the PubMed Database, ensuring that all search results are up-to-date. While it is possible to download the entire database and store it locally, the effort required to do so is prohibitive (Oliver et al., 2004), and we found the PubMed servers to be fast enough to allow direct queries.

Use of the tool has also revealed a few limitations of PubMed QUEST, some of which can not easily be addressed. When searching for authors in aggregate, it is essential to ensure that the names provided are not only spelled correctly (it is very easy to miss a misspelling among a list of 100 names) but that the correct initials are used as well. Correct initials are especially important for common names. If two names (last name as well as initials) are the same, then there is currently no way to distinguish between them. As a result, if those individuals belong to different programs, the publications will count towards each program.

**Figure 2.**
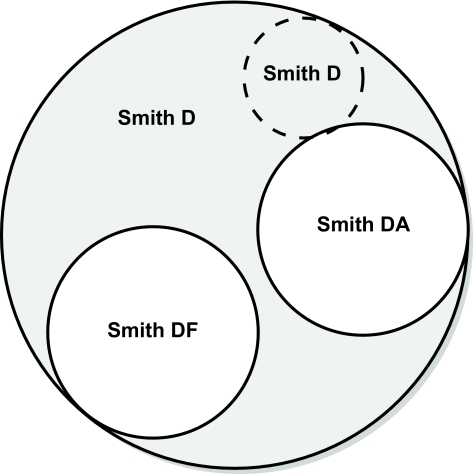
A diagram illustrating the complexity of searching for publications by an author. A search using the term “Smith D” will return all results from the large circle. A search for “Smith DA” will return only the small white circle to the right. However, if an author has listed their name as “Smith DA” in some publications and “Smith D” in others, then searching with the term “Smith DA” could result in some publications being excluded (denoted using the circle with dashed lines).

It is ideal to use the most specific name possible when conducting a search as this will limit the number of citations attributed incorrectly to a specific individual. However, if an author has published papers using an inconsistent set of initials, then there is a greater risk of either including papers which do not belong or excluding papers which do belong. Please see Figure 2 for more details.

Our tool is not able to identify publications for which authors contributed as part of a multi-institutional collaboration but where the primary institution was not our own. This is a byproduct of limiting our search to our own institution.

These problems described could be resolved for future publications if PubMed were to assign a unique identifying number to each author (Bohne-Lang & Lang, 2005). Confusion over attributing publications to authors who share names could then easily be resolved. Without such a mechanism in place there will likely always remain some ambiguity without human intervention from someone familiar with an author’s publications. Although this would require a large initiative involving the registration of users and the maintenance of an accurate and up-to-date database, an online process could be instituted where each author had to have a unique ID before submitting an article. This would aid in keeping track of an author’s work even if they changed their names or the institution to which he or she belonged.

As a result of the limitations described, it is best to assume that PubMed QUEST provides an estimate of the number of publications as opposed to an absolute list. Our experience, however, has shown that program leaders have also had difficulty providing a “true” list given the large numbers of authors for which they need to summarize. Additionally, the ability to apply standard rules to marking both intra- and inter-programmatic publications has greatly simplified this complex task and allowed for a consistent and reliable listing of such publications for all of our core programs. The ease with which the tool can be used is also an advantage since it now allows more users to search PubMed using complex queries without the difficulty of learning the intricacies of PubMed itself.

We believe that this tool has served us well and it may also be of interest to other institutions. With little effort, and a minimal amount of maintenance to keep the lists updated, this tool could easily be ported to other institutions.

## References

[b1-cin-02-79] Bohne-Lang A, Lang E (2005). Do we need a Unique Scientist ID for publications in biomedicine?. Biomed Digit Libr.

[b2-cin-02-79] Ding J, Berleant D (2005). MedKit: a helper toolkit for automatic mining of MEDLINE/PubMed citations. Bioinformatics.

[b3-cin-02-79] Gall C, Brahmi FA (2004). Retrieval comparison of EndNote to search MEDLINE (Ovid and PubMed) versus searching them directly. Med Ref Serv Q.

[b4-cin-02-79] Lin SM, McConnell P, Johnson KF (2004). MedlineR: an open source library in R for Medline literature data mining. Bioinformatics.

[b5-cin-02-79] NCBI 2005aPubMed Overview. Accessed 24 October 2005. URL: http://www.ncbi.nlm.nih.gov/entrez/query/static/overview.html

[b6-cin-02-79] NCBI 2005bHelp Manual: Table 1. Search Field Descriptions and Tags. Accessed 24 October 2005. URL: http://www.ncbi.nlm.nih.gov/books/bv.fcgi?rid=helppubmed.table.pubmedhelp.T37

[b7-cin-02-79] Oliver DE, Bhalotia G, Schwartz AS (2004). Tools for loading MED-LINE into a local relational database. BMC Bioinformatics.

